# Anemia among Women Attending Antenatal Care at the University of Gondar Comprehensive Specialized Referral Hospital, Northwest Ethiopia, 2017

**DOI:** 10.1155/2018/7618959

**Published:** 2018-10-09

**Authors:** Wubet Worku Takele, Amare Tariku, Fasil Wagnew Shiferaw, Amare Demsie, Wondale Getinet Alemu, Degefaye Zelalem Anlay

**Affiliations:** ^1^Department of Community Health Nursing, School of Nursing, College of Medicine and Health Sciences, University of Gondar, Gondar, Ethiopia; ^2^Department of Human Nutrition, Institute of Public Health, College of Medicine and Health Sciences, University of Gondar, Gondar, Ethiopia; ^3^Department of Nursing, College of Medicine and Health Sciences, Debre Markos University, Ethiopia; ^4^Department of pediatrics and Child Health Nursing, School of Nursing, College of Medicine and Health Sciences, University of Gondar, Gondar, Ethiopia; ^5^Department of Psychiatry, school of medicine, College of Medicine and Health Sciences at the University of Gondar, Ethiopia; ^6^Department of Community Health Nursing, School of Nursing College of Medicine and Health Sciences, University of Gondar, Gondar, Ethiopia

## Abstract

**Background:**

In Ethiopia, prenatal anemia is a major public health concern affecting both the health of the woman and babies. The World Health Organization recommends to conduct repeated prevalence studies concerning prenatal anemia . However, there is no recent evidence on the magnitude of the prenatal anemia. Therefore, the aim of this study was to determine the prevalence and the associated factors of prenatal anemia among women attending the Antenatal Care Clinic at the University of Gondar Referral Hospital.

**Methods:**

A facility-based cross-sectional quantitative study was conducted among 362 participants from June 03-July 08, 2017, at the University of Gondar Comprehensive Specialized Hospital, Northwest Ethiopia. The systematic random sampling technique was employed. Structured interviewer administered questionnaire was used. Human Immunodeficiency Virus (HIV) screening was conducted. Nutritional status of the participants was assessed. Blood sample was collected by capillary tube . Intestinal parasite was examined by stool wet mount test. HIV serostatus was detected. Anemia was defined as hemoglobin concentration below 11 g/dl. The multivariable logistic regression model was employed to identify associated factors and to control the possible effects of confounders.

**Result:**

The prevalence of anemia was 22.2% (95% CI: 18.11, 27.1%). The highest odds of anemia were observed among pregnant women with family size of >five [AOR = 3 (95% CI: 1.03, 8.65)], unprotected water source users, [AOR = 4.09 (95% CI: 1.75, 9.55)], HIV infected [AOR = 2.94(95% CI: 1.37, 6.35)], and multigravida women [AOR = 3.5 (95% CI: 1.35, 9.17)].

**Conclusion and Recommendations:**

The prevalence of anemia among pregnant women attending the University of Gondar Referral Hospital was a moderate public health problem. Unprotected water source, large family size, Human Immunodeficiency Virus infection, and repeated pregnancies were factors that predicted anemia. Thus, prevention of Human Immunodeficiency Virus infection, family planning utilization, and accessing pure water are recommended.

## 1. Background

Pregnancy is the critical period in which many preventable nutritional deficiencies happened secondary to unmet nutrient requirements. According to the World Health Organization (WHO) report, prenatal anemia is defined as hemoglobin concentration of below 11g/dl[1]. Maternal mortality, impairment of fetal neurocognitive development, and behavioral disorders are some of the sequelae of prenatal anemia. Furthermore, anemia during pregnancy is a risk factor for poor pregnancy outcomes, such as low birth weight (LBW), prematurity [2–5], stillbirth [6, 7], and intrauterine growth restriction [8–10]. In addition, it exposes women to cardiovascular disease [11] and fetal vital organ hypoxia [9]. Another deleterious effect of anemia is thyroid hormone dysfunction following reduction of the activity of thyroid peroxidase (TPO) enzyme [12, 13].

Globally, prenatal anemia is a critical hematological disorder affecting 32.4 million pregnant women of which 46.3% are found in Africa with 1.5% severe anemia [14]. A study in India showed that 74.7% pregnant women were anemic [15]. According to the 2016 Ethiopian National and Demographic Health Survey (EDHS) 23% [16] of reproductive age women were suffering from anemia. The same figure was reported by a national study in 2011 [17]. Several studies in Ethiopia revealed that nutritional anemia is a public health concern in all regions of the country with high proportions, like 36.6% [18], 39.9% [19], and 19.7% [20].

In Ethiopia, some of the multifactorial causes of anemia are low meal frequency, consumption of poorly diversified diets, and repeated pregnancies [20, 21]. Poor income, large family size, undernutrition [15, 19, 22, 23], and short birth interval (<24 months) are the other factors contributing to prenatal anemia [22]. Moreover, the likelihood of developing prenatal anemia was high among women who were not supplemented with iron folate, were illiterate, had history of infectious diseases (malaria, HIV/AIDs), and exposed to Hookworm infection [23–26]. The odds of prenatal anemia were high among women who have restrictive dietary behavior (less food portion size and fasting) and chew khat [27]. Furthermore, food taboos and adhering to some cultural malpractices lead to anemia [28]. The WHO has proposed reducing anemia by 50% by the year 2025 [29]. To achieve this target, the organization recommended repeated prevalent studies capable of pointing out the magnitude of anemia and its contributing factors [30]. Though Ethiopia has exerted many efforts for the reduction of anemia through designing National Nutrition Program (NNP), Health Sector Development Program VI (HSDP), and essential nutrition action with a special focus on maternal and child nutrition, the magnitude of anemia decreased only steadily.

Therefore, this study was conducted to determine the prevalence and the determinants of anemia. The outcome could be useful for nutrition program managers and local decision makers working on maternal and child health projects.

## 2. Methods and Materials

### 2.1. Study Design and Setting

The institution based cross-sectional study was carried out to assess the prevalence and associated factors of anemia among pregnant women attending the Antenatal Care Clinic at the University of Gondar Comprehensive Specialized Referral Hospital from June 03 to July 08, 2017. The hospital is found in Gondar town which serves for more than five million people. The town is located northwest Ethiopia at an altitude of 2133 m (6890 ft) above the sea level with an average rainfall of 116 mm per year.

### 2.2. Study Population, Inclusion, and Exclusion Criteria

All pregnant women who were receiving ANC at the University of Gondar Referral Hospital during the data collection period were the study population. Pregnant women who were physically available at ANC clinic at the time of data collection were included in the study, whereas mentally incapacitated women were excluded.

### 2.3. Sample Size Determination

The sample size was estimated using Epi Info version 7 by considering the following statistical assumptions: prevalence of anemia (16.4%), a study conducted in the same area earlier [25], 95% level of confidence, 4% margin of error, and 10% nonresponse rate. Thus, the final study participants turned out to be 362.

### 2.4. Sampling Technique and Procedure

The systematic random sampling technique was used. During the study the average number of pregnant women attending ANC clinic in the preceding one and half month was 1245. A sampling fraction of 3(K^th^ = N/n) was calculated by dividing the total number of pregnant women receiving ANC in the last one and half month by the sample size. Following the selection of the first study participant using the lottery method, every third woman of the initial participants was included. Nonetheless, the next participant was considered whenever the selected one did not fulfill the inclusion criteria or was unwilling to participate after informed consent.

### 2.5. Data Collection Procedures and Tools

The data were collected by using a face to face interviewer-administered questionnaire. The questionnaire was developed by reviewing the 2016 EDHS report and variety of literature [16, 19, 20, 31]. The tool contained, but is not limited to, sociodemographic characteristics, wealth index tools, obstetric history, blood tests, stool examination, HIV test, anthropometry measurement, and dietary practice questions. Prior to the data collection pretest was conducted. Accordingly, some language and typographic corrections were made. The data were collected by trained nurses and midwives.

#### 2.5.1. Assessment of Hemoglobin Concentration

Blood samples were collected by finger prick using lancet and added to capillary tubes. The blood sample was analyzed using hematological machines** SYXMEX KX-21 hematology analyzer (Sysmex Corporation, Kobe, Japan)**. The WHO standard definition was used to ascertain anemia; women with hemoglobin concentration below 11 g/dl were considered as anemic [1]. The severity of anemia was defined as hemoglobin concentration of <7 g/dl, 7-9.9 g/dl and 10-10.9 g/dl as severe, moderate, and mild anemia, respectively.

#### 2.5.2. Assessment of Parasitic and HIV Infection Test

A fresh stool was collected using clean stool cups and the stool wet mount test was prepared using saline within 30 minutes of sample collection [32]. Accordingly, the presence of hook worm infection was examined microscopically.

HIV test was performed by using the nationally standardized algorism in that initially women's serostatus was checked by the Beijing one-tie (reaction of a drop of blood and buffer waited for 30 minutes); reactive women for the first test were rechecked by unigold (reaction of 2 drops of blood and a drop of buffer waited for 10 minutes), and reactive women for this test were reported as HIV positive. However, if there was discordance result between first and second tests, serostatus confirmation was made using Vikia (reaction of 3 drops of blood and a drop of buffer waited for 30 minutes) whose result was taken as reported.

#### 2.5.3. Maternal Nutritional Status and Dietary Diversity Measurement

The nutritional status of the participants was determined by Mid-Upper Arm Circumference (MUAC) between the shoulder and (olecranon) and the tip of the elbow (acromion process) of the nondominant hand using a nonstretchable and nonelastic tape. Thereafter, woman who had less than 22 cm MUAC was categorized as underweight [33].

Woman's dietary diversity was assessed by 10 standardized lists of food items recommended by the Food and Nutrition Technical Assistance (FANTA) 2016 Version. Using a 24-hour food recall method, participants were interviewed to report the food items consumed among the listed food groups in the past 24 hours, including day and night. In cases of mixed dishes, the dish was divided into individual food items and then categorized into the respective food groups. Respondent's dietary diversity score was computed together. Minimum dietary diversity score (MDDS) was dichotomized and coded as 0 and 1 for respondents who consumed less than five group items and greater than or equal to five items, respectively. Finally, the MDDS was categorized as adequate dietary diversity if the woman consumed five and more food items [34]. Accordingly the proportion of MDD was reported.

#### 2.5.4. Measurement of Household Wealth Status

Household wealth status was determined by using items adapted from the Ethiopian Demographic Health and Survey (EDHS) 2016 [16] which included number of livestock, availability of agricultural land, quantity of cereal products, money/birr available in the bank, and kind of materials the house was built from. Continuous variables were categorized in a range and appropriate weight (between 0 and 1) was given. For categorical variables having greater than two categories, again values were between 0 and 1. After transforming all continuous and categorical variables between 0 and 1, Principal Component Analysis (PCA) was performed. Variables with a communality value of greater than 0.5 were included in the final analysis. After the summation of eligible components, variables were corrected to be in the loading factor between three and negative three to control the outliers. Finally, the score was ranked as five quintiles, namely, richest, rich, middle, poorer, and poorest.

### 2.6. Data Analysis

The collected data were coded and entered into EP INFO Version 7 and analyzed using STATA/SE Version 14. Descriptive statistics like frequency, percentage, and the measure of central tendency with the corresponding measure of dispersion were used for the presentation of demographic data and other variables. Tables, graphs, and other data summary mechanisms were also used for data presentation. The binary logistic regression model was fitted to identify factors associated with anemia. The bivariable analysis was done, and all variables which had a p value of < 0.2 were entered into the multivariable analysis model to identify the significant variables as well as control the possible effect of confounders. Model goodness of fit test was checked by Hosmer-and Lemeshow test (p value = 0.22). Multicollinearity was checked by using the variance inflation factor (VIF), and it was confirmed that no multicollinearity was detected. Finally, the variables which had independent correlations with anemia were identified on the basis of the Adjusted Odds Ratio (AOR) with a 95% CI and p value less than 0.05.

*Ethics Approval*. Ethical clearance was obtained from the Institutional Review Board of the University of Gondar. Permission letter was obtained from the University of Gondar Referral Hospital. Furthermore, after a thorough discussion and explanations of the purpose, benefits, and possible risks of the study, written informed consent was secured from participants. For participants who were unable to read and write, the information was read to them in the presence of witnesses and their agreement was confirmed by pressing their finger prints in the prepared informed consent format. Women aged below 18 years were allowed to participate considering that they were emancipated minors. The information confidentiality was maintained by avoiding personal identifiers, such as names and using only numerical codes. The questionnaire was kept safe by locking in cabinet throughout the whole process. During data collection, for any woman who had medical findings, such as anemia and malnutrition, necessary treatment and nutrition education were given. For women newly diagnosed with HIV, after a detailed counseling the treatment was initiated since the current HIV treatment guideline of the country recommends test and treat irrespective of the CD4 count. Likewise, women with intestinal parasitic infestation were treated with appropriate medication.

## 3. Result

### 3.1. Sociodemographic Characteristics

A total of 362 participants took part in the study with a response rate of 92%. The mean (±standard deviation) age of the participants was 26.7 (±5.8) years. The age of about 55% of participants ranged between 25 and 34 years. The vast majority (89.7%) of the pregnant women lived in urban area. Nearly, two-thirds (62%) of the participants have attended college and above. More than half (56.3%) of the participants had two and below family members in the household. The majority of participants (91%) accessed water from protected sources (Table 1).

### 3.2. Obstetric Characteristics

Over half (56.6%) of the pregnant women were on third trimester. Two-thirds (66%) were primigravida. Most (92.4%) reported that they had no history of bleeding during pregnancy (Table 2)

### 3.3. History of Morbidity from Infectious Diseases

**Figure 1:**
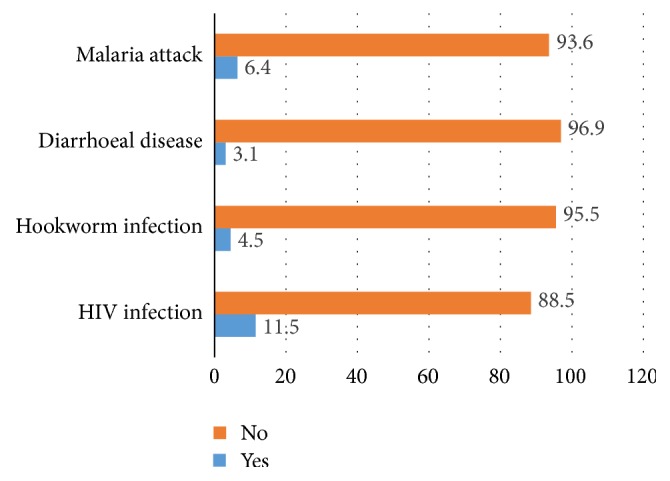
Comorbidity status of pregnant women with infectious disease attending ANC at the University of Gondar Comprehensive and Specialized Referral Hospital.

Regarding comorbid conditions, about 6.4% and 3.1% of the respondents were infected with malaria and diarrheal diseases in the last one month, respectively, whereas 11.5% of the pregnant women were living with the Human Immunodeficiency Virus (HIV) (Figure 1).

### 3.4. Dietary and Nutrition Related Characteristics

Regarding dietary characteristics, 72.6% of participants reported that they consumed vegetable three and more times weekly. Besides, a bit more than half (53.1%) of the pregnant women received iron supplementation in the current pregnancy. Moreover, about 84.9% pregnant women never received deworming medications. The current finding demonstrated that 20.4% of pregnant women were underweight, while 28.9% consumed diversified diet. Furthermore, all pregnant women reported that they never chewed Khat and smoked cigarettes in their life-time.

### 3.5. Prevalence of Anemia

The overall prevalence of anemia among pregnant women attending the University of Gondar Comprehensive Hospital was 22.2% (95% CI: 18.11, 27.1%), and the mean hemoglobin concentration was 12 mg/dl (±5.4). Regarding the severity of anemia, about 18.7%, 1.5%, and 2.1% of women were severely, moderately, and mildly anemic, orderly.

### 3.6. Factors Associated with Anemia

In the bivariable model, eight variables at a p value below 0.2 entered into the multivariable regression model. Nevertheless, only five independent variables were found statistically significant, namely, source of drinking water, parity, gravidity, HIV infection, and household family size.

The likelihood of having anemia among participants with household members of five and greater was threefold higher [AOR = 3 (95% CI: 1.03, 8.65)] as compared to less than two and lesser household members.

The odds of anemia among subjects who accessed drinking water from unprotected sources were four times higher [AOR = 4.09(95% CI: 1.75, 9.55)] compared to tap water users.

In the current study it was identified that repeated pregnancy correlated with anemia. The odds of anemia among pregnant women who gave single births decreased by 60% [AOR = 0.4 (95% CI: 0.15, 0.96)] as compared to multiparous women. Likewise, the highest likelihood of anemia was noted among multigravida women as 3.5 times higher [AOR = 3.5 (95% CI: 1.35, 9.17)] compared to women who were primigravida. The study also revealed that the odds of experiencing anemia increased by threefold among pregnant women with HIV infection [AOR = 2.94 (95% CI: 1.37, 6.35)] compared to their counterparts (Table 3).

## 4. Discussion

There is by far little knowledge about the magnitude and determinant factors of anemia in the study area. Therefore, this study was aimed at determining the burden of anemia and associated factors among pregnant women. 

This finding exhibited that the magnitude of prenatal anemia was 22.2% deemed as a moderate public health problem. This indicates that still prenatal anemia is continuing to be a problem in this segment of population. Unless otherwise necessary strategies are not planned and implemented, the problem will be worsened and pose a potential threat to the success of reduction of maternal and child mortality in the country. This finding is in parallel with the Ethiopian Demographic and Health Survey (EDHS) national report and other previous studies elsewhere in Ethiopia [17, 20, 31]. Similarly, the result of this study is comparable with studies shown in other countries [35, 36].

However, the finding is lower than multiple studies reported in other parts of Ethiopia which reported that the prevalence is ranging from 30.5% to 43.9% [19, 22, 24, 27]. It is reasonable that the improvement of ANC coverage, prenatal iron supplementation, involvement of woman in decision-making process, and decreasing the burden of maternal undernutrition in the country could favor the reduction of the magnitude [16]. This is supported by earlier studies showing that antenatal care follow-up, enhancing women empowerment, and good maternal nutritional status prevent anemia [37]. The provision of micronutrient (folic acid and iron), nutrition education, and other related cares during ANC visit might reduce the risk of developing anemia. Moreover, the study demonstrated that most (89.7%) of the participants lived in urban areas which is believed to have the lesser probability of encountering anemia related to better awareness, hygiene, and sanitation [31].

A higher likelihood of anemia was observed among women who had greater than five household members than those who had two family members and less. This finding is concordant with that of a study conducted in Southern Ethiopia [19]. In some areas of Ethiopian culture, women and children in large families are supposed to feed leftover foods which is poorly qualified food. Besides, as number of family members raises, unless the wealth status of the family is improved food insecurity, meal frequency, and dietary diversity will be a great concern. Despite the fact that many studies reported the relationship between family size and anemia still the problem demands further investigations.

The result of this study revealed that the odds of anemia among unprotected water consumers increased fourfold compared to that among protected water source users. This finding is suggesting that there should be accessibility of pure water for the community. This finding is supported by studies done elsewhere [38, 39]. This could be due to the fact that poor environmental sanitation may lead to a frequent diarrheal attack and mal-absorption problems which result in environmental enteropathy/environmental enteric dysfunction (EED) and subsequently causes poor micronutrient absorption in the human intestine.

Strong association was noted among women with anemia and HIV/AIDs infection. This result is highlighting that a strong work has to be done on the prevention and treatment HIV/AIDs among pregnant women. The finding is consistent with previous studies [25, 26, 40]. The cause of anemia among people living with HIV/AIDs is diverse and primarily due to the reduction of red blood cells (RBCs) production [41]. Likewise, alterations in immune activation markers (cytokines) and iron metabolism are attributed to low hemoglobin concentration status [42]. Secondly, the already formed RBCs by the virus were destructed. Thirdly, hookworm and other intestinal infections were detected among people living with HIV/AIDs associated with incompetent immune system [43, 44], which are responsible for anemia. Moreover, vitamin A deficiency is more prominent in HIV/AIDs infected patients [45] which leads to poor iron absorption which is believed to ameliorate iron absorption in the intestine [46]. Studies outlined that vitamin A has a great role in facilitating the release of stored iron from the liver, enhancing erythropoietin activity and iron absorption [47, 48].

Another important factor positively associated with prenatal anemia was repeated pregnancy. Multigravida women had 3.5 times more likelihood of facing anemia than primigravida. This result is congruent with what was reported by a study done earlier [19]. It is well known that repeated birth/high fertility behavior is a risk factor for micronutrient depletion owing to high demand and frequent blood loss during delivery [49] which ultimately affect micronutrient storage.

Though the current study hardly showed the association between iron supplementation in the current pregnancy and anemia, other former studies done elsewhere demonstrated the presence of association [23, 24]. The disparity between our finding and the results of other works may be explained in terms of the small sample size we used and other methodological variations.

Incorporating other very important predictors' anemia like HIV test, stool examination through laboratory tests and determining nutritional status of the women make this study stronger. However, the study is not free from some limitations. For instance, recall and social desirability bias linked with dietary practices are among the shortcomings of the study. It would have been worthy if iron storage status, RBCs, and other micronutrient (e.g., vitamin B-12 and vitamin A) profiles had been determined. Lastly, it would have been great if the current status of malaria infection was diagnosed through laboratory test, rather than ascertaining the infection through history. We have interviewed the women on whether they have experienced malaria infection in the previous one month or not.

## 5. Conclusion

All in all, prenatal anemia among women attending ANC clinic at the University of Gondar Referral Hospital, Northwest Ethiopia, was described as a moderate public health problem. Repeated pregnancy, family size, the source of water, and HIV infection were factors which correlated with anemia. Hence, promotion of family planning, nutrition education, prevention of HIV infection and due attention to people living with HIV/AIDs, environmental sanitation, and making safe water accessible to the community are strongly recommended. In addition, further investigation into the cause of anemia in this segment of population is recommended for future researchers.

## Figures and Tables

**Table 1:** Sociodemographic characteristics of pregnant women attending ANC at the University of Gondar Referral Hospital (n = 332).

**Variables **	**Number **	**Percentage**
**Age **		
15-24	109	32.8
25-34	181	54.5
35-49	42	12.7
**Residence **		
Urban	298	89.7
Rural	34	10.3
**Ethnicity**		
Amhara	309	93.1
Tigre	23	6.9
**Educational status**		
Unable to read and write	48	15.0
Primary	78	25.0
College and above	204	60.0
**Occupation **		
Governmental employee	91	27.4
Merchant	60	18.0
House wife	181	54.6
**Family size **		
≤2	187	56.3
3-5 members	101	30.5
	44	13.2
**Wealth index**		
Richest	66	19.8
Rich	67	20.2
Middle	66	19.8
Poorer	67	20.2
Poorest	66	19.8
**Toilet **		
Yes	305	91.9
No	27	8.1
**Source of drinking water **		
Tap water	302	91.0
Pond/river	30	9.0
**Utilization of impregnated bed net**		
Yes	137	41.3
No	195	58.7

**Table 2:** Obstetric characteristics of pregnant women attending ANC at the University of Gondar Referral Hospital (n = 332).

Trimester	**Number **	**Percentage **
First trimester	48	14.5
Second trimester	96	28.9
Third trimester	188	56.6
**Parity **		
Nulliparous	153	46
Primiparous	90	27.2
Multiparous	89	26.8
**Gravidity **		
Primi-gravida	219	66
Multigravida	113	34
**ANC visit **		
New	141	42.5
Repeat	191	57.5
**Duration of menstrual bleeding**		
≤5	175	52.7
>5 days	125	47.2
**Bleeding during pregnancy**		
Yes	307	92.4
No	25	7.6
**Family planning utilization**		
Yes	272	81.9
No	60	18.1

**Table 3:** Factors associated with anemia among pregnant women attending ANC services at the University of Gondar Referral Hospital, Northwest Ethiopia, 2017 (n = 332).

**Variables **	**Anemia status **		**Crude odds ratio (95% CI)**	**Adjusted odds ratio (95% CI)**
	**Anemic **	**Not anemic**		
**Source of water**				
Tap	60	242	1:00	1:00
Pond/river	14	16	3.5(1.63, 7.6)	**4.09(1.75, 9.55)** ^**∗∗∗**^
**Vegetable consumption in a week**				
≥3 times	49	192	0.67 (0.38, 1.17)	0.66 (0.33,1.34)
<2 times	25	66	1:00	1:00
**Parity **				
Nulliparous	35	118	0.64 (0.35, 1.16)	0.5 (0.17, 1.73)
Para one	11	79	0.3 (0.13,0.65)	**0.4 (0.15, 0.96)**
Multiparous	26	61	1:00	1:00
**Gravidity**				
Primi-gravida	43	176	1:00	1:00
Multigravida	31	82	1.54 (0.9, 2.63)	**3.5 (1.35, 9.17)** ^**∗∗**^
**ANC visit **				
New	26	115	1:00	1:00
Repeated	48	143	0.67 (0.39, 1.15)	0.5 (0.32, 1.05)
**HIV infection**				
Yes	16	22	2.9 (1.46, 5.9)	**2.94 (1.37, 6.35)** ^**∗∗**^
No	58	236	1:00	1:00
**Family size **				
≤2	38	149	1:00	1:00
3-5 members	19	82	0.9 (0.49, 1.67)	1.77(0.68, 4.57)
>5 members	17	27	2.46 (1.22, 4.98)	**3 (1.03, 8.65)** ^**∗**^
**Fruit consumption in a week**				
≥3 times	48	96	1.09 (0.63, 1.87)	1.6 (0.81, 3.16)
< 2 times	26	162	1:00	1:00

1:00 reference category.

^**∗∗∗**^P value < 0.001.

^**∗∗**^P value < 0.01.

^**∗**^P value < 0.05.

## Data Availability

The data used to support the findings of this study are included within the article.

## Abbreviations

DDS: Dietary diversity score
EDHS: Ethiopian Demographic Health Survey
FANTA: Food And Nutrition Technical Assistance
HIV: Human Immunodeficiency Virus
HSDP: Health Sector Development Program
MUAC: Mid-Upper Arm Circumference
PCA: Principal Component Analysis
VIF: Variance Inflation Factor
WHO: World Health Organization.
